# A Deletion Downstream of the *CHCHD7* Gene Is Associated with Growth Traits in Sheep

**DOI:** 10.3390/ani10091472

**Published:** 2020-08-21

**Authors:** Hongwei Xu, Haixia Li, Zhen Wang, Ayimuguli Abudureyimu, Jutian Yang, Xin Cao, Xianyong Lan, Rongxin Zang, Yong Cai

**Affiliations:** 1 College of Life Science and Engineering, Northwest Minzu University, Lanzhou 730030, China; xuhongwei@xbmu.edu.cn (H.X.); 280051210@xbmu.edu.cn (A.A.); yangjutian@xbmu.edu.cn (J.Y.); 278081666@xbmu.edu.cn (X.C.); 2 Science Experimental Center, Northwest Minzu University, Lanzhou 730030, China; 3 College of Animal Science and Technology, Northwest A&F University, Yangling 712100, China; lhx9529@163.com (H.L.); wangzhenid@126.com (Z.W.); lanxianyong79@nwsuaf.edu.cn (X.L.)

**Keywords:** *CHCHD7* gene, downstream deletion mutation, growth traits, *PLAG1* gene, sheep breeds

## Abstract

**Simple Summary:**

The genes *CHCHD7* and *PLAG1* are located on the same growth-related major quantitative trait locus of sheep. *PLAG1* affects sheep growth, but no corresponding studies have been conducted on *CHCHD7*. However, polymorphisms in the *CHCHD7* gene are associated with carcass weight and muscle formation in cattle, body height in cattle and humans, and weaning weight in Duroc pigs. In this study, the mathematical expectation method was used to analyze an 8-bp deletion mutation located downstream of the *CHCHD7* gene in 2350 individuals from seven sheep breeds. The associations between wild-type and deletion genotypes and growth traits in Tan sheep were also analyzed. The 8-bp deletion locus was significantly associated with body length (*p* = 0.032), chest depth (*p* = 0.015), and chest width (*p* = 0.047) of Tan sheep. Additionally, wild-type genotype carriers were more numerous than those heterozygous for the deletion genotype. Thus, the genotyped 8-bp deletion downstream of the *CHCHD7* gene may be associated with growth and development traits in sheep.

**Abstract:**

In sheep, the coiled-coil-helix-coiled-coil-helix domain containing 7 (*CHCHD7*) gene and the pleiomorphic adenoma gene 1 (*PLAG1*) are on the same growth-related major quantitative trait locus, positioned head-to-head approximately 420 bp apart on chromosome 9. *PLAG1* affects sheep growth, but the effects of *CHCHD7* have not been determined. In this study, an 8-bp deletion downstream of *CHCHD7* was analyzed in 2350 sheep from seven breeds. The associations between the deletion and growth traits of Tan sheep were also determined. Both genotypes (homozygous wild-type and heterozygous) for the 8-bp deletion were found in Tan (TS), Luxi Blackhead (LXBH), Small-Tail Han (STHS), and Lanzhou Fat-Tail (LFTS) sheep. However, there were no polymorphic sites for the mutation in Hu (HS), Sartuul (SS), and Australian White (AUW) sheep. In TS, LXBH, STHS, and LFTS sheep, the deletion genotype was less frequent than the wild-type genotype, and the allele frequencies of the deletion variant were 0.007 (TS), 0.011 (LBXH), 0.008 (STHS), and 0.010 (LFTS). The 8-bp deletion was significantly associated with body length (*p* = 0.032), chest depth (*p* = 0.015), and chest width (*p* = 0.047) in Tan sheep. Thus, the 8-bp deletion downstream of the *CHCHD7* gene might be associated with growth and development traits of sheep.

## 1. Introduction

Sheep provide meat, fur, and other valuable products. In particular, the meat, which is nutritious and tender, has significant economic value. In China, sheep breeding has a crucial role in regional economic development, but some shortcomings remain in the production of sheep meat [1,2]. Traditional breeding methods to improve the growth traits of sheep are expensive and time consuming. Therefore, the use of marker-assisted selection in breeding has been widely studied as an alternative [3,4]. Insertions and deletions (indels) are a particular type of genetic marker that can be used as a molecular marker in the genetic selection of livestock [3,4]. Therefore, the indels located within or close to potentially relevant genes in sheep need to be analyzed [5,6].

Genome-wide association studies reveal that polymorphisms of the coiled-coil-helix-coiled-coil-helix domain containing 7 (*CHCHD7*) gene are associated with cattle carcass weight [7] and body height and muscle formation [8], human body height [9,10], and Duroc pig weaning weight [11]. In sheep, *CHCHD7* and pleiomorphic adenoma gene 1 (*PLAG1*), another important candidate gene widely reported to influence animal growth traits, are positioned head-to-head approximately 420 bp apart on chromosome 9 [12]. The ~25 Mbp major pleiotropic quantitative trait locus (QTL) on bovine chromosome 14 affects numerous growth and development characteristics in *Bos taurus*, which are also attributed to two functional variants between the *PLAG1* and *CHCHD7* genes [13]. These studies indicate that *CHCHD7* and *PLAG1* might coregulate the growth and development of sheep. Some deletion mutations of *PLAG1* significantly affect sheep growth [14,15]. Recently, Wu et al. [15] found two deletion variants within the sheep *PLAG1* gene, which are located in a growth-related major QTL associated with body weight and morphometric traits. However, the relationships between polymorphisms in the *CHCHD7* gene and growth traits of sheep have not yet been examined. The *CHCHD7* gene is highly homologous in many species [16]. In this study, the *CHCHD7* gene, which has great potential to affect animal growth traits, was used to investigate this relationship between polymorphism and seven sheep breeds. The linkage disequilibrium (LD) between a deletion mutation downstream of the *CHCHD7* gene and two reported mutations in the *PLAG1* gene was also analyzed in order to explore the co-segregation patterns between polymorphisms of these two genes.

Polymerase chain reaction (PCR) and the mathematical expectation (ME) method are widely used in the detection and analysis of mutations [3,17]. For low-frequency mutations, compared with PCR, which requires scanning samples one by one, ME is an efficient and feasible method for screening [3]. There are five steps in the ME approach. First, the minimum allele frequency (MAF) of the analyzed mutation is estimated by using random sampling from the available population. Then, based on the population size and estimated MAF, the reaction times (the amplification times required for samples to complete genotyping) are calculated on the basis of particular formulae [3], and the sample size in one group is determined as the minimum reaction time. Based on the most optimal sample size in one group, samples are tested by dividing them into several groups, and each group is genotyped. If only one specific genotype is displayed in a given group, then all samples in that group belong to the same genotype. If a group shows different genotypes, then each sample from that group needs to be re-examined using PCR. Finally, all samples are genotyped [3].

The ME strategy has been widely used in medical censuses to study rare diseases, because it requires less time and money and is easily performed [3,17]. Because scanning numerous samples one by one requires much time and effort, in this study, to identify genotypes of sheep populations quickly and at low cost, PCR was used to analyze the frequency of deletion genotypes, and ME was used to estimate the deletion genotype in individual sheep. The main aim of this study was to determine the segregation pattern of an 8-bp deletion downstream of the *CHCHD7* gene in sheep populations as well as to analyze the association between the polymorphic site and growth traits in sheep, which could provide useful information for marker-assisted selection.

## 2. Materials and Methods

### 2.1. Ethics Statement

The Institutional Animal Care and Use Committee of Northwest A&F University (IACUC-NWAFU) approved all experiments conducted in this study. The research was conducted in full compliance with animal welfare policies and legal guidelines.

### 2.2. Animal Samples and Data Collection

A total of 2350 sheep from the following seven sheep breeds were sampled in this study: Tan (TS; *n* = 907, containing 276 samples from Wu et al. [15]), Luxi Blackhead (LXBH; *n* = 629), Small-Tail Han (STHS; *n* = 190), Lanzhou Fat-Tail (LFTS; *n* = 49), Hu (HS; *n* = 201), Sartuul (SS; *n* = 48), and Australian White (AUW; *n* = 326). The sheep were reared in the Chinese provinces of Ningxia (TS), Shandong (LXBH), Gansu (STHS and LFTS), and Henan (HS); Zhawkhan Province, Mongolia (SS); and Tianjin City, China (AUW). Among the 907 Tan sheep, there were 458 rams and 449 ewes. All sheep were healthy adults of similar ages. Among the 629 LXBH sheep, there were 234 lambs and 395 adults. All animals were allowed free access to feed and water under standard conditions. Data on growth traits of Tan sheep breed were collected, including body weight, body height, body length, height at the hip cross, chest depth, chest width, paunch girth, cannon circumference, and hip width [18]. In this study, no growth data were recorded for SS and AUW sheep. Growth traits of Tan sheep individuals are shown in Supplement 1.

### 2.3. Isolation and Use of Sheep Genomic DNA

Genomic DNA was collected from the ear tissue of seven breeds (preserved in 70% alcohol and stored at −80 °C) via a high-salt and phenol–chloroform extraction protocol [19]. The quality and purity of each DNA sample were measured by a Nanodrop 1000 (Thermo Fisher Scientific Inc., Wilmington, DE, USA). Then, DNA samples were diluted with ddH_2_O to the standard concentration of 50 ng/µL and stored at −20 °C for subsequent genotyping.

### 2.4. Primer Design, PCR Amplification, and Genotyping

The 8-bp deletion mutation (NC_040260.1: g36219994_3622000delGTTACAAG, rs593501397) was located 2758 bp downstream of the *CHCHD7* gene in sheep. Primers were designed to amplify this mutation by PRIMER PREMIER 5 software (PRIMER Inc., Canada). The forward and reverse primers were 5′-TGGCCTTGACAGACACATCC-3′ and 5′-CCTGCAGAGCTTCCCTTTCT-3′, respectively, and the amplicon size was 156 bp. Touchdown PCR cycling was run with an initial denaturation for 5 min at 95 °C; 18 cycles of denaturation at 94 °C for 30 s, annealing at 68 °C for 30 s (with a decrease of 1 °C per cycle), and extension at 72 °C for 20 s; another 23 cycles of denaturation at 94 °C for 30 s, annealing at 50 °C for 30 s, and extension at 72 °C for 20 s; and a final extension at 72 °C for 10 min, with a final cooling at 4 °C. The genotypes were detected by simultaneous sequencing (Sangon Biotech, Shanghai, China) and 3.5% agarose gel electrophoresis at a constant voltage of 120 V for 60 min.

**Figure 1:**
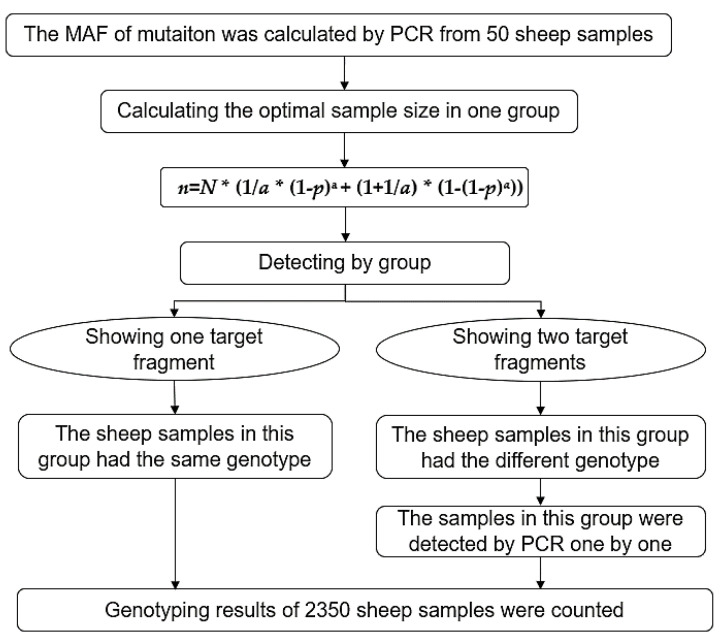
Graphical explanation of the ME method.

First, 50 randomly selected individuals were genotyped to determine the estimated MAF, and on the basis of this result, the decision was made to use the ME method. The ME method is explained graphically in Figure 1.

### 2.5. Linkage Disequilibrium Analysis with Two Deletions in the PLAG1 Gene

According to Wu et al. [15], the *PLAG1* gene has two deletion mutations in the Tan sheep population (30-bp and 45-bp deletions). Tan sheep samples (*n* = 276) were genotyped, including 151 rams and 125 ewes. The primers of the PCR amplification were designed and the PCR amplification procedure was conducted as reported previously [15]. In this study, the genotyping results of the two mutation sites within the *PALG1* gene were obtained in 276 Tan sheep. Then, the LD between the 8-bp deletion mutation downstream of the *CHCHD7* gene and the two known mutations in the *PLAG1* gene was analyzed to investigate the co-segregation patterns of those mutations between the two genes. The results of the LD analysis were obtained from the online website (http://analysis.bio-x.cn/myAnalysis.php). The case of D’ = 1 and r^2^ = 1 is complete LD. Values of D’ < 1 and r^2^ > 0.33 indicate that the complete ancestral LD has been disrupted, but strong LD remains, whereas values of D’ < 1 and r^2^ < 0.33 indicate weak LD.

### 2.6. Statistical Analyses

The genotype frequencies, allele frequencies, and MAF were analyzed on the SHEsis platform (http://analysis.bio-x.cn) [20,21]. Population parameters, including homozygosity (Ho), heterozygosity (He), and the effective number of alleles (Ne), were also analyzed [22]. The Kolmogorov–Smirnov test was used to verify whether the data conformed to the normal distribution by SPSS 23.0 version (SPSS Inc., Chicago, IL, USA). Association analyses of the 8-bp deletion locus downstream of the *CHCHD7* gene and growth traits in the different breeds were performed with Mann–Whitney U tests (SPSS version 23.0) [17,23]. 

## 3. Results

### 3.1. Genotyping of 8-bp Deletion Downstream of the CHCHD7 Gene

**Figure 2:**
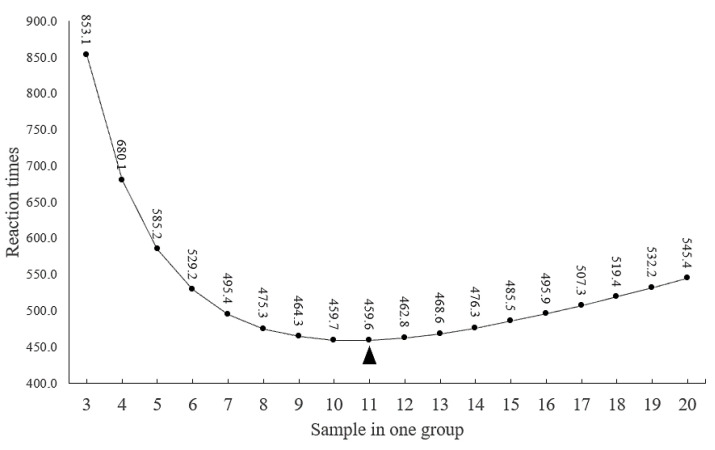
Identification of all sheep populations with reaction times when the number of samples in one group ranged from 3 to 20. “▲” stands for the most optimal sample size of one mixed group.

**Figure 3:**
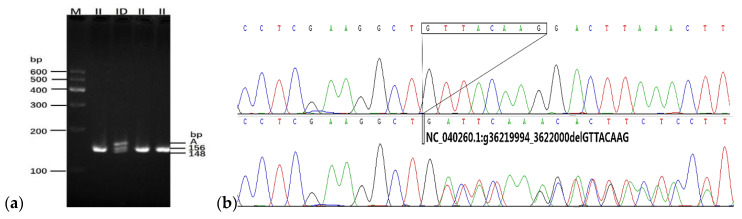
The electrophoresis pattern (**a**) and the sequence diagram (**b**) of the 8-bp deletion locus located downstream of the sheep *CHCHD7* gene. II—wildtype genotype; ID—deletion genotype. The M represents the Marker I; the A represents the non-target fragment called heteroduplex.

The genotyping of the 50 sheep samples one by one revealed only one heterozygous deletion genotype, indicating that the MAF of this deletion site in the sheep population was approximately 0.01. Accordingly, this mutation site was considered to be a low-frequency mutation. In this case, the ME method was appropriate to genotype the sheep population. According to Yang et al. and the analytic website (http://www.msrcall.com/DALMcall.aspx), the following equation (Equation (1)) [3] was used to calculate the reaction times:

*n* = *N* × (1/*a* × (1 − *p*)^a^ + (1 + 1/*a*) × (1 − (1 − *p*)*^a^*)) (1)

where *n* is the reaction time (the amplification time required for 2350 sheep samples to complete genotyping), *N* is the total sample size (*N* = 2350), *a* is the sum of a group (*a* = 3 to 20), and *p* is the MAF (*p* = 0.01). The predicted reaction times are given in Figure 2. When the number of samples in a group was 11, the lowest reaction time (459.6) was used to detect all 2350 individuals. Therefore, to identify genotypes in the present study, 11 sheep samples were mixed for each group. Ultimately, the genotypes of all 2350 individuals were successfully detected through a reaction time of 460. According to the genotyping results, only four of the seven breeds (TS, LXBH, STHS, and LFTS) showed polymorphic sites for the 8-bp deletion (Figure 3). The deletion mutation was not polymorphic in HS, SS, and AUW sheep (Table 1). The genotyping results of the seven sheep breeds are shown in Supplement 1.

### 3.2. Genetic Parameter Analysis of the 8-bp Deletion Downstream of the Sheep CHCHD7 Gene

The genotype and allele frequencies of the 8-bp deletion downstream of the *CHCHD7* gene are shown in Table 1. The deletion genotype was in fewer TS, LXBH, STHS, and LFTS sheep than the wild-type one. The frequencies of the deletion allele were 0.007 in TS, 0.011 in LXBH, 0.008 in STHS, and 0.010 in LFTS sheep. Deletion polymorphism was not found in HS, SS, and AUW sheep. In addition, Ho, He, and Ne were calculated separately for all seven breeds. All genotyping results of the seven sheep breeds are given in Supplement 1.

### 3.3. Analysis of the Association between the 8-bp Deletion and Growth Traits in Tan Sheep

**Figure 4:**
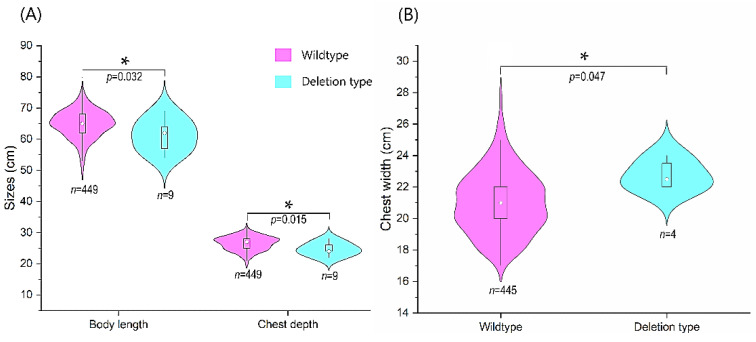
Association between the 8-bp deletion downstream of sheep *CHCHD7* gene and body measurement traits in Tan sheep (**A**), rams; (**B**), ewes. * represents a significant difference. *n* is the number of samples.

The Kolmogorov–Smirnov test indicated that the growth data of Tan sheep did not fit a normal distribution (Table S1 in Supplement 2), so the Mann–Whitney U test was used to analyze the effects of the different genotypes on the growth traits of Tan sheep. The deletion genotype was significantly associated with body length (*p* = 0.032) and chest depth *(p* = 0.015) in 458 TS rams. The deletion was also significantly associated with chest width (*p* = 0.047) in TS ewes (Table 2). The effects of the different genotypes of this mutation on the growth traits of sheep are expressed via a violin plot (Figure 4).

### 3.4. Linkage Disequilibrium Analysis between the 8-bp Deletion Downstream of the CHCHD7 Gene and Two Deletions in the PLAG1 Gene

The LD between the 8-bp deletion mutation downstream of the *CHCHD7* gene and the two reported variations in the *PLAG1* gene was analyzed. In the genetic analysis of linkage disequilibrium on three deletions (8-bp downstream of the *CHCHD7* gene and 30-bp and 45-bp deletions of the *PLAG1* gene) in Tan sheep, the case of D’ < 1 and r^2^ < 0.33 indicated that the LD was weak. Thus, the 8-bp deletion downstream of the *CHCHD7* gene and the two deletion variations within the *PLAG1* gene were not in strong linkage. The LD diagram is given in Figure S1 (Supplement 2). To summarize, the 8-bp variation downstream of the *CHCHD7* gene may not play a role in sheep growth by LD with the two deletion mutations in the *PLAG1* gene.

## 4. Discussion

In this study, an 8-bp deletion downstream of the *CHCHD7* gene was examined in 2350 individuals from seven sheep breeds by using PCR and ME. The deletion mutation occurred with low frequency, justifying the use of the ME method. For low-frequency mutations, compared with the PCR method of scanning samples one by one, ME is an effective and practical method for screening [3].

In the present study, the genotypes of this locus varied by breed. The genotyping result of each sample of the seven sheep breeds is shown in Supplement 1. Only the wild-type homozygous genotype was detected in HS, SS, and AUW sheep. However, both genotypes (wild-type and deletion genotype) were found in TS, LXBH, STHS, and LFTS sheep. This difference might be due to differences in breeding, including differences in geographic locations of breeds and various environmental factors [24], or perhaps, the number of individuals with heterozygous deletion genotypes was too small to detect in HS, SS, and AUW sheep. In previous studies, nontarget products were heteroduplexes [25], and heteroduplexes were only in individuals heterozygous for the mutation [26]. The associations between the 8-bp deletion in the downstream region of *CHCHD7* and the growth traits of TS sheep were also analyzed. The growth data of Tan sheep did not fit a normal distribution (Table S1), so a Mann–Whitney U test was used to analyze the effects of different genotypes on the growth traits of Tan sheep. Depending on the outcome, the 8-bp deletion mutation could affect sheep growth traits. In addition, carriers of the wild-type genotype were more abundant than individuals with the deletion genotype in Tan sheep (Table 1).

The *CHCHD7*–*PLAG1* gene region on bovine chromosome 14 is related to the height of European cattle breeds [27]. In addition, several QTNs (Quantitative Trait Nucleotides) that affect height and carcass weight have been found in the *CHCHD7*–*PLAG1* gene region [27,28,29]. *PLAG1* regulates several growth factors, including *IGF2*, which affects growth and body weight [30,31]. *PLAG1* itself, in which deletion mutations cause significant changes in sheep weight [14,15], is a dominant gene affecting the height of humans and cattle [32]. The QTNs in the *CHCHD7*–*PLAG1* intergenic region are connected with male carcass traits and birth weight in cattle [7,16,33]. A QTL with a significant effect on bovine stature was found using LD mapped to the *CHCHD7– PLAG1* intergenic region [34], suggesting that *CHCHD7* and *PLAG1* genes have a specific relationship in regulating the growth and development of organisms. Hence, the relationship between *CHCHD7* and *PLAG1* genes in sheep should be examined further.

Wu et al. [15] previously detected two deletion variations within the sheep *PLAG1* gene located in a growth-related major QTL associated with body weight and morphometric traits. In the current work, the genotyping results of the two mutation sites within the *PLAG1* were obtained in Tan sheep. Then, the LD between an 8-bp deletion mutation downstream of the *CHCHD7* gene and the two mutations in the *PLAG1* gene was analyzed in order to explore the relationship between the two genes. The 8-bp deletion downstream of the *CHCHD7* gene was not strongly linked with the two deletion variations in the *PLAG1* gene (Figure S1). Therefore, the 8-bp deletion site downstream of the *CHCHD7* gene may not play a role in sheep growth by co-segregation with the two deletion mutations within the *PLAG1* gene. Although no LD relationship between these three deletion mutations in the *CHCHD7* and *PLAG1* genes was found in this study, the relationship between *CHCHD7* and *PLAG1* genes in sheep still merits further study. Moreover, numerous studies have reported on the effects of mutations in noncoding regions on the growth and development of domestic animals [30,35]. In recent years, the candidate genes that affect growth traits have been extensively studied. For example, the mutation of the *IGF2BP1* gene in the 3’ untranslated region can significantly affect caprine growth traits [4,36]. Therefore, the specific mechanism of action of the 8-bp deletion deserves to be explored further.

## 5. Conclusions

In summary, an 8-bp, low-frequency deletion mutation downstream of the *CHCHD7* gene was analyzed in sheep, and it was weakly but significantly associated with growth traits of Tan sheep. Therefore, the 8-bp deletion mutation might be a potential DNA marker for marker-assisted selection in sheep breeding.

## Figures and Tables

**Table 1:** Genetic parameter of 8-bp deletion of *CHCHD7* gene in seven sheep breeds.

Breeds	Sizes	Genotypic Frequencies		Allelic Frequencies		Population Parameters			
		Wildtype	Deletion	Wildtype	Deletion	Ho	He	Ne	MAF
TS	907	0.986 (*n* = 894)	0.014 (*n* = 13)	0.993	0.007	0.986	0.014	1.014	0.007
LXBH	629	0.978 (*n* = 615)	0.022 (*n* = 14)	0.989	0.011	0.978	0.022	1.023	0.011
STHS	190	0.984 (*n* = 187)	0.016 (*n* = 3)	0.992	0.008	0.984	0.016	1.016	0.008
LFTS	49	0.980 (*n* = 48)	0.020 (*n* = 1)	0.990	0.010	0.980	0.020	1.021	0.010
HS	201	1.000 (*n* = 201)	0 (*n* = 0)	1.000	0	1.000	0	1.000	0
SS	48	1.000 (*n* = 48)	0 (*n* = 0)	1.000	0	1.000	0	1.000	0
AUW	326	1.000 (*n* = 326)	0 (*n* = 0)	1.000	0	1.000	0	1.000	0

Note: TS—Tan sheep; LXBH—Luxi Blackhead Sheep; STHS—Small Tail Han sheep; LFTS—Lanzhou Fat Tail Sheep; HS—Hu Sheep; SS—Sartuul sheep; AUW—Australian White sheep. Ho—homozygosity; He—heterozygosity; Ne—effective allele numbers; MAF—minimum allele frequency.

**Table 2:** Association between the 8-bp deletion locus of the sheep *CHCHD7* gene and growth traits in Tan sheep (Mann–Whitney U test).

Gender	Traits	Genotypes (LSM ± SE)		*p*-Values
		Wildtype	Deletion Type	
Rams	BW (kg)	33.71 ± 0.11 (*n* = 449)	33.03 ± 0.58 (*n* = 9)	0.256
	BH (cm)	65.04 ± 0.15 (*n* = 449)	62.67 ± 1.86 (*n* = 9)	0.237
	**BL (cm)**	**64.85 ^a^ ± 0.21** (*n* = 449)	**61.22 ^b^ ± 1.70** (*n* = 9)	**0.032**
	HHC (cm)	62.52 ± 0.14 (*n* = 449)	60.56 ± 1.89 (*n* = 9)	0.287
	PG (cm)	78.78 ± 0.19 (*n* = 449)	78.33 ± 1.00 (*n* = 9)	0.717
	**CD (cm)**	**26.54 ^a^ ± 0.11** (*n* = 449)	**24.66 ^b^ ± 0.68** (*n* = 9)	**0.015**
	CW (cm)	20.74 ± 0.09 (*n* = 449)	20.56 ± 0.60 (*n* = 9)	0.649
	CC (cm)	8.14 ± 0.03 (*n* = 449)	8.33 ± 0.14 (*n* = 9)	0.171
Ewes	BW (kg)	33.33 ± 0.10 (*n* = 445)	33.48 ± 0.42 (*n* = 4)	0.900
	BH (cm)	63.74 ± 0.15 (*n* = 445)	61.75 ± 1.11 (*n* = 4)	0.164
	BL (cm)	65.12 ± 0.22 (*n* = 445)	64.00 ± 1.35 (*n* = 4)	0.493
	HHC (cm)	61.94 ± 0.14 (*n* = 445)	61.00 ± 0.91 (*n* = 4)	0.525
	PG (cm)	79.83 ± 0.18 (*n* = 445)	79.25 ± 2.10 (*n* = 4)	0.649
	CD (cm)	26.42 ± 0.10 (*n* = 445)	25.50 ± 0.65 (*n* = 4)	0.316
	**CW (cm)**	**21.01 ^b^ ± 0.09 (*n* = 445)**	**22.75 ^a^ ± 0.48 (*n* = 4)**	**0.047**
	CC (cm)	8.03 ± 0.03 (*n* = 445)	8.00 ± 0.46 (*n* = 4)	0.937

Note: LSM—least squared means; SE—standard error; BW—body weight; BH—body height; BL—body length; HHC—height at the hip cross; CD—chest depth; CW—chest width; PG—paunch girth; CC—cannon circumference. The row in bold represents that there is a significant difference in different genotypes of the same traits. a and b represent significant differences. The significance level was 0.05.

## Supplementary Materials

The following are available online at https://www.mdpi.com/2076-2615/10/9/1472/s1. The genotyping results of this 8-bp mutation in different sheep populations were shown in Supplement 1. Distribution diagram of different growth traits of Tan sheep were shown in Table S1 of Supplement 2. Genetic analysis of linkage equilibrium on three deletion mutations of Tan sheep were shown in Figure S1 of Supplement 2.
