# Supplementary material for: A Deletion Downstream of the CHCHD7 Gene Is Associated with Growth Traits in Sheep
Source: Animals (Basel). 2020 Aug 21;10(9):1472. doi: 10.3390/ani10091472 (PMC7552293; doi:10.3390/ani10091472)
Supplement: Supplementary file 1 [file animals-10-01472-s001.zip › Supplement materials/Supplement 2.docx]

**Table S1.** Distribution diagram of different growth traits of rams and ewes in Tan sheep.

| Rams | Distribution diagram | *p*-value | Ewes | Distribution diagram | *p*-value |
| --- | --- | --- | --- | --- | --- |
| BW (kg) | 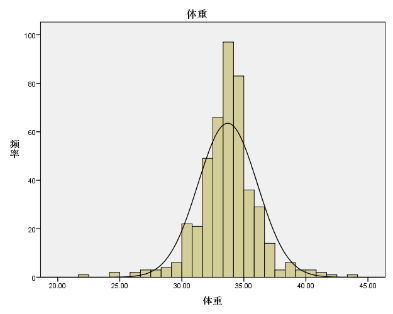 | *p*＜0.05 | BW (kg) | 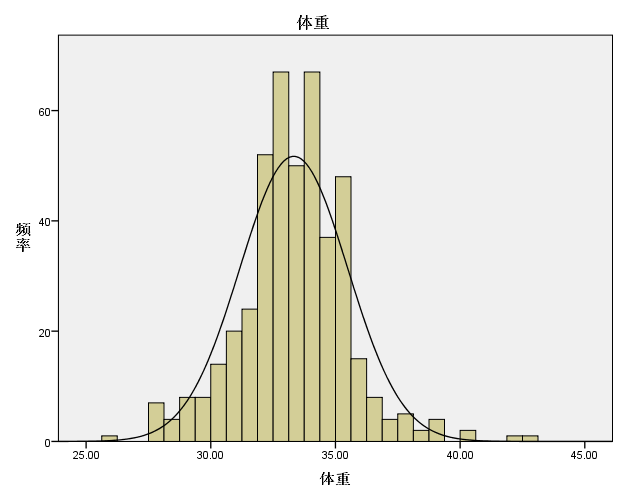 | *p*＜0.05 |
| BH (cm) | 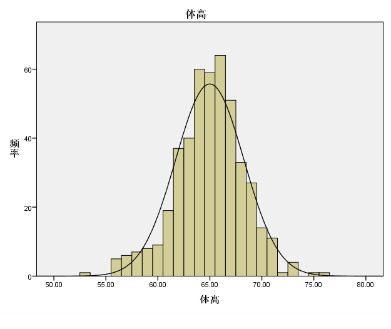 | *p*＜0.05 | BH (cm) | 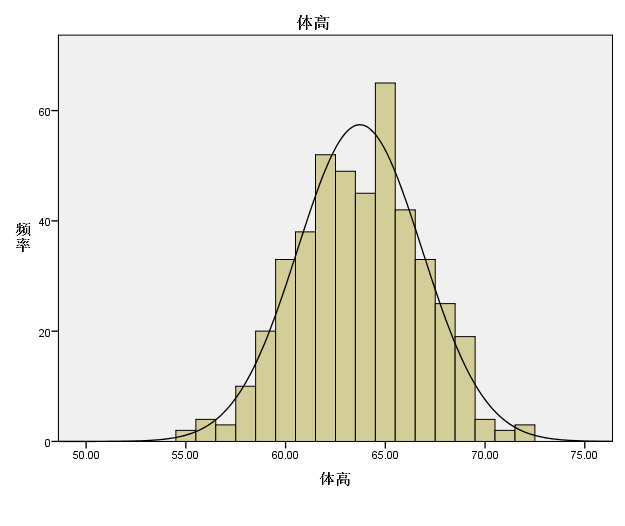 | *p*＜0.05 |
| BL (cm) | 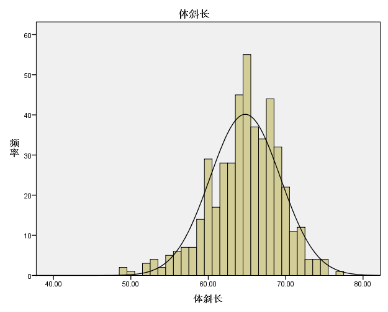 | *p*＜0.05 | BL (cm) | 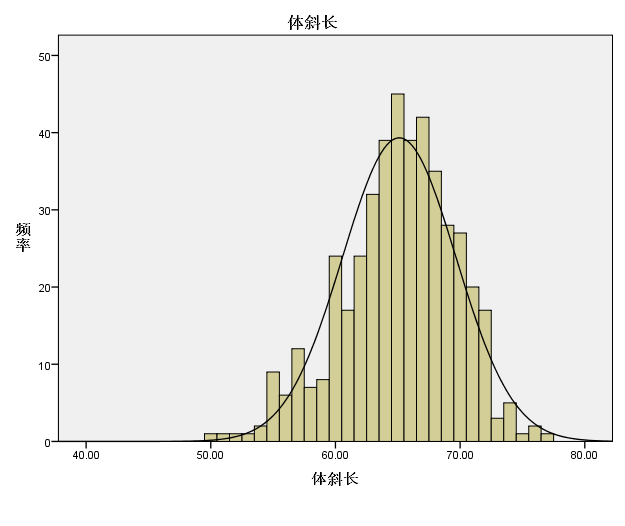 | *p*＜0.05 |
| HHC (cm) | 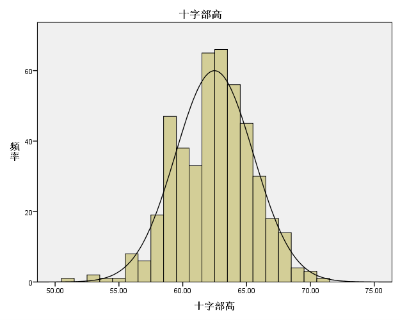 | *p*＜0.05 | HHC (cm) | 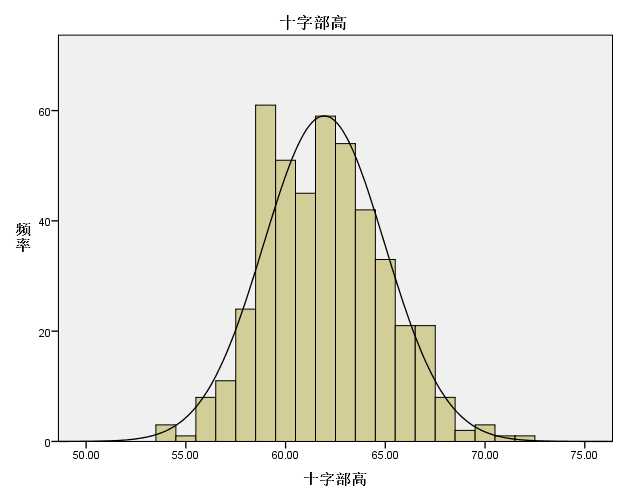 | *p*＜0.05 |
| PG (cm) | 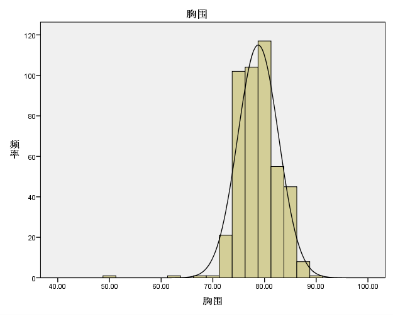 | *p*＜0.05 | PG (cm) | 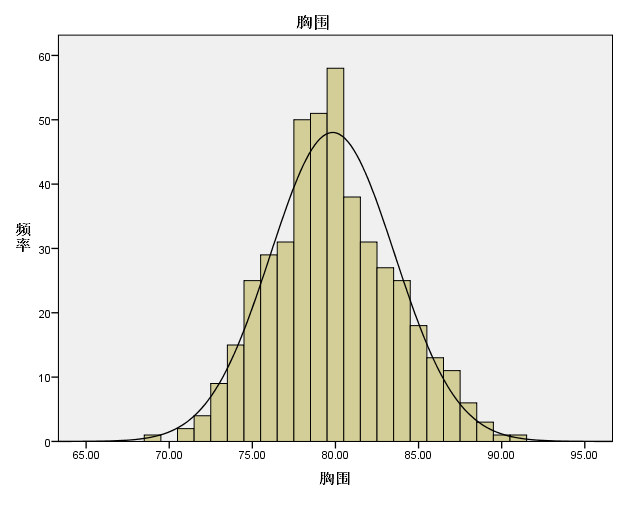 | *p*＜0.05 |
| CD (cm) | 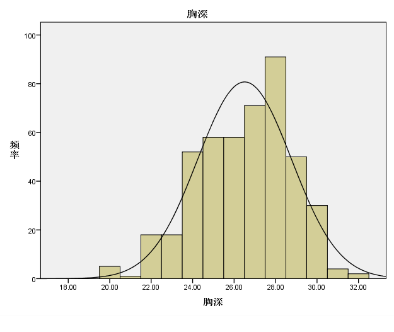 | *p*＜0.05 | CD (cm) | 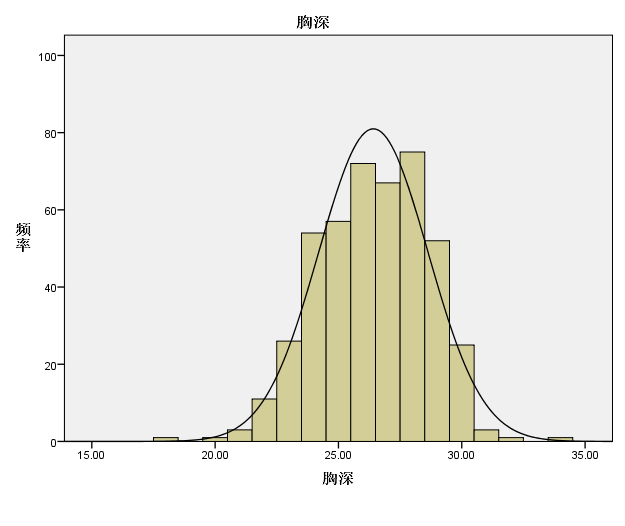 | *p*＜0.05 |
| CW (cm) | 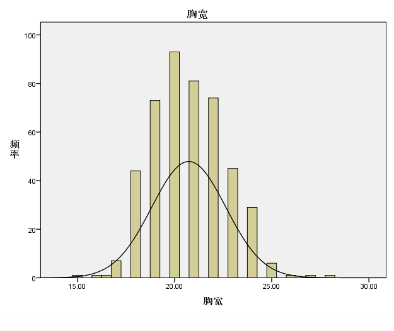 | *p*＜0.05 | CW (cm) | 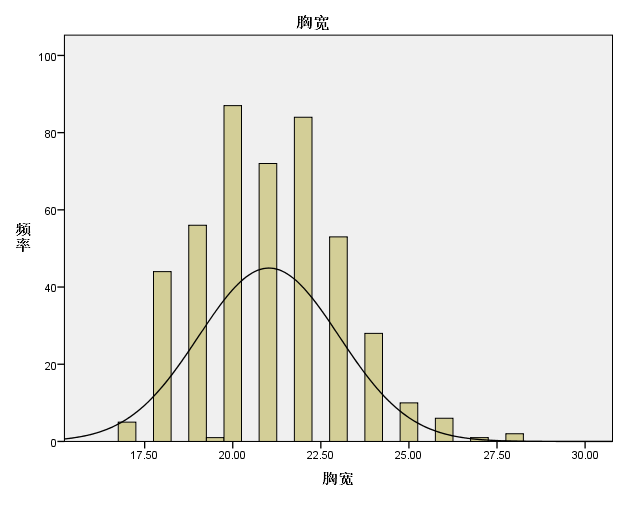 | *p*＜0.05 |
| CC (cm) | 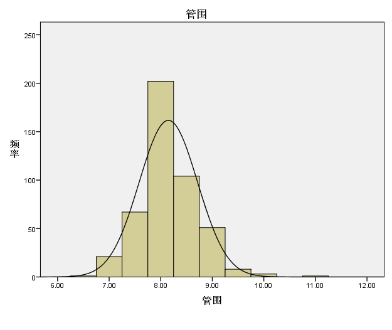 | *p*＜0.05 | CC (cm) | 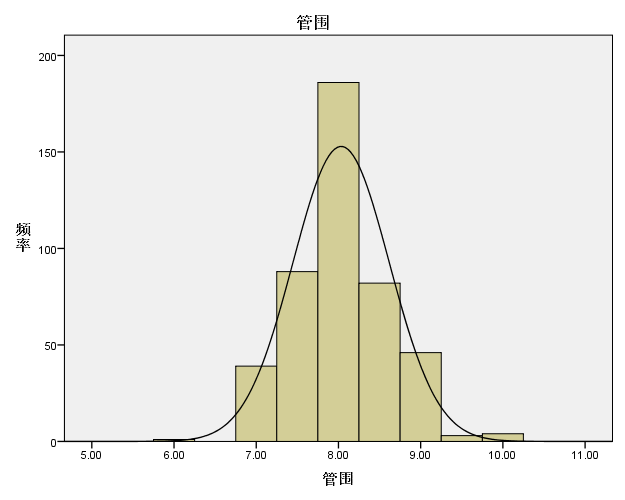 | *p*＜0.05 |

Note: Kolmogorov-Smirnov test is used to determine whether it conforms to normal distribution. Null hypothesis, growth data accord with normal distribution, *p*＜0.05 reject null hypothesis. BW, body weight; BH, body height; BL, body length; HHC, height at the hip cross; CD, chest depth; CW, chest width; PG, paunch girth; CC, cannon circumference.


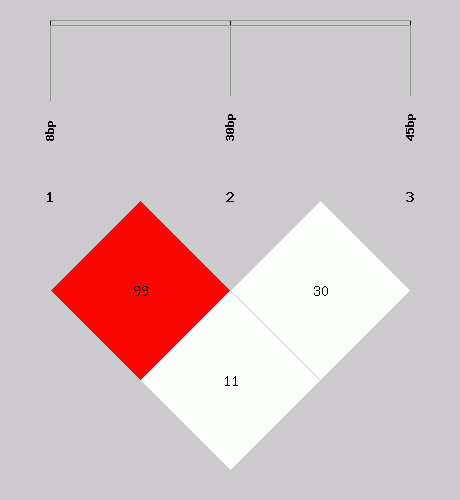

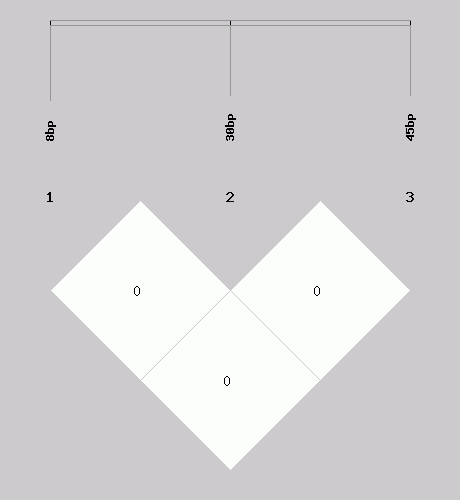


**Figure S1.** Genetic analysis of linkage equilibrium on three deletion (8 bp of *CHCHD7* gene, 30 bp and 45 bp deletion of *PLAG1* gene) in Tan sheep. left, D’ value; right, r^2^ value.
